# K-Selection as Microbial Community Management Strategy: A Method for Improved Viability of Larvae in Aquaculture

**DOI:** 10.3389/fmicb.2018.02730

**Published:** 2018-11-14

**Authors:** Olav Vadstein, Kari J. K. Attramadal, Ingrid Bakke, Yngvar Olsen

**Affiliations:** ^1^Department of Biotechnology and Food Science, Norwegian University of Science and Technology (NTNU), Trondheim, Norway; ^2^Department of Biology, Norwegian University of Science and Technology (NTNU), Trondheim, Norway

**Keywords:** microbial ecology, dysbiosis, r-selection, microbial control, fish larvae, antibiotics, host–microbe, mutualism

## Abstract

Aquaculture has the potential to become a major food supplier in a world with an increasing human population, and increased consumption of fish will likely have positive health implications. For marine aquaculture, the production of high quality juveniles is a bottleneck. Survival until the juvenile stage is typically as low as 10–15% for many species, which indicates suboptimal rearing conditions. Substantial evidence indicates that the poor performance and viability of larvae is largely due to detrimental larvae-microbiota interactions. This emphasises the need for microbial management strategies in the cultivation of marine fish larvae. Disinfection and probiotics are the most studied microbial management methods so far. However, most studies on these methods overlooked the role of mutualistic relationships between microbes and hosts, and have not proposed or examined methods steering toward such relationships. Based on ecological theory and a number of experiments, we find support for the hypothesis that current practise in aquaculture generally selects for r-strategic, opportunistic microbes, which results in detrimental host–microbiota interactions. Thus, the challenge is to develop technology and methods for microbial management at the ecosystem level that creates a K-selected microbial community, and by this mean select against r-strategic opportunists. Here we summarise experiments done during 25 years and with marine larvae of five different species showing that: (1) K-selection strategies result in different water microbiota with less opportunists, (2) this influences the microbiota of the fish larvae, and (3) the larvae cultivated in water inhabited by a K-selected microbiota perform better. Improved performance of larvae includes improved appetite, earlier onset of and faster growth, increased survival, and increased robustness to stress. K-selection as a method for management of the microbial community is a robust approach that allows steering of host–microbiota interactions in larviculture toward mutualism. It could also be applicable for young stages of other domesticated animals. Our review illustrates that a change from a “beat-them” to a “join-them” strategy for microbial management in larval rearing can lead to a more sustainable aquaculture industry.

## Introduction

Aquaculture is the fastest growing food industry worldwide (Duarte et al., 2009), and marine fish is an important source of protein, essential long-chain omega 3 fatty acids, and micronutrients for the increasing human population (Beveridge et al., 2013; Thilsted et al., 2016). Furthermore, marine aquaculture has the potential to significantly contribute to the amount of food needed for the 9.2 billion humans expected to inhabit Earth by 2050 (Duarte et al., 2009). However, the production of high quality juveniles is a bottleneck in marine aquaculture (Planas and Cunha, 1999; Vadstein et al., 2013). For many species, survival of larvae on industry scale is typically as low as 10–15%, and this is an indicator of suboptimal rearing conditions. Substantial evidence indicates that detrimental larvae-microbiota interactions can be a major explanation for the poor performance and viability of the larvae (see below and Vadstein et al., 2013). Accordingly, microbial management strategies in larval rearing are needed. The problems experienced in marine aquaculture resemble those observed for early stages of farmed animals in agriculture, which in several cases have resulted in abuse of antibiotics (Gustafson and Bowen, 1997; Cabello, 2006; Aminov, 2010). Thus, the problem with poor viability is not restricted to aquaculture.

There has been a substantial increase in the understanding of microbe-host interactions in animals during the last two decades. This has resulted in new knowledge on the functional roles of such microbes, including healthy development of the and a wide range of diseases not considered to be infection related, like diabetes and autism (e.g., Kanther and Rawls, 2010; Sekirov et al., 2010; McFall-Ngai et al., 2013; Lerner et al., 2017). The importance of microbe-fish interactions in host development and health are less studied, but this area of research is also rapidly progressing (see Kanther and Rawls, 2010; Vadstein et al., 2018; Vestrum et al., 2018b). This rapid progress is largely due to a revolution in molecular and imaging methods (Vestrum et al., 2018a). Studies on fish are important not only from an applied perspective (aquaculture), but also to increase our knowledge on fish, as this is a diverse key group in the early evolution of jawed vertebrates that started with prehistoric fish about 500 million years ago (Vandepoele et al., 2004).

In this review, we first summarise data showing that detrimental larvae-microbe interactions are a main cause of the poor viability of cultured marine larvae. Secondly, as a basis for understanding and resolving this problem, we analyse the first feeding system from a microbial ecology perspective. Thirdly, this analysis is used to propose a hypothesis on microbial community management by K-selection. Finally, we use mainly our own published results to test our K- selection hypothesis, and predictions from this hypothesis, in a variety of rearing systems and a range of species. The present paper reviews 30 years of research using the concept of r/K-strategists (Pianka, 1970) and pioneer/mature-community theory (Odum, 1971) to analyse microbial communities in the rearing of marine larvae and to steer them toward mutualistic relationships. Furthermore, this review evaluates ideas, hypotheses, and microbial management methods we proposed 25 years ago (Vadstein et al., 1993b).

## The Symptoms and Possible Explanations

The symptoms observed just before and during crashes in larval rearing are generally agreed upon in the research community and in the industry, and include the following observations: the larvae do not start to eat properly, many larvae that eats grow slowly, their normal development may stop at a critical stage, abrupt mortality is normal, and the reproducibility between replicate rearing tanks is poor. The short version of these observations is poor performance of larvae and lack of reproducibility, and consequently predictability. The magnitude of the problem depends on species and rearing technology for the given species, but typically the survival is 3–10% at metamorphosis at an early stage of domestication. It is interesting to note that people rearing ornamental fish experience the same problems (e.g., Moorhead and Zeng, 2010). In addition, for farmed animals, mortality problems are mainly observed at early life stages (Heier et al., 2002; Alonso-Spilsbury et al., 2007). This has resulted in abuse of antibiotics in both aquaculture and agriculture (Gustafson and Bowen, 1997; Cabello, 2006; Romero et al., 2012).

Some representatives of the aquaculture industry claim that a stable survival of 10 to 15% is sufficient for economic sustainability. We will argue from a biological perspective that this is too simplistic. There is no reason why most of the eggs (>80%) produced from a well-nourished fish broodstock would die, and therefore we claim that survival around 50% or lower is an indicator of suboptimal rearing conditions. Moreover, this suboptimal start in life may have long lasting consequences, as there is substantial evidence from several animals, including humans, that suboptimal conditions during early life are critical for later performance. For humans, a well-documented example is insufficient supply of long-chain omega 3 fatty acids in prematurely born children. It is generally recommended that foetus and neonates should receive n-3 long chain poly-unsaturated fatty acids (PUFA) in amounts that are sufficient for optimal long-term development of visual and cognitive capacity. Oils rich in n-3 long chain PUFA given during pregnancy can also reduce the risk for early premature birth (Koletzko et al., 2008). Furthermore, evidence from epigenetics emphasises that a suboptimal start in life may be even more critical than previously anticipated. Studies in Sweden have demonstrated a relationship between human obesity, diabetes, and life expectancy to whether the children mothers or grandmothers had experienced starvation in early life (Pembrey et al., 2014). These negative changes in health were due to epigenetic changes. Thus, in aquaculture, a continuous effort is made to improve viability of the fish, efforts that will also lead to improvement of both animal welfare and economical sustainability.

For a long time, inadequate egg quality and larval nutrition, especially of n-3 long chain fatty acids, were considered the main causes of the problems observed during larval rearing (Brooks et al., 1997; Rainuzzo et al., 1997). Even though these are essential and critical factors, they cannot explain all the symptoms described above. First, insufficient nutrition normally does not result in abrupt change in performance. Second, and even more important, the lack of reproducibility between full sibling groups treated equally in early stages (feed and environmental conditions) cannot be due to poor egg quality or poor nutrition. Motivated by the lack of adequate explanations, microbial-related conditions was hypothesised as the cause of the above mentioned symptoms by Vadstein et al. (1993b).

During the last 20–25 years, a substantial number of studies using different approaches supported this hypothesis. They strongly indicate that detrimental fish-microbe interactions are a key factor causing poor performance and low reproducibility during first feeding of marine larvae. These studies include the use of antibiotics (Vadstein et al., 1993b; Munro et al., 1994, 1999; Verner-Jeffreys et al., 2004; Sørensen et al., 2014), surface disinfection of eggs (Salvesen and Vadstein, 1995; Salvesen et al., 1997; Grotmol and Totland, 2000), bacteria free larvae (Munro et al., 1995; Dierckens et al., 2009; Forberg et al., 2011), experiments with manipulation of the microbiota in the environment (Makridis et al., 2000a,b; Forberg et al., 2012), manipulation of the microbiota of live feed (Munro et al., 1999; Olsen et al., 2000), and correlations at the individual level between composition of the microbiota and growth rate of larvae (Forberg et al., 2016; Trinh et al., 2017).

**FIGURE 1:**
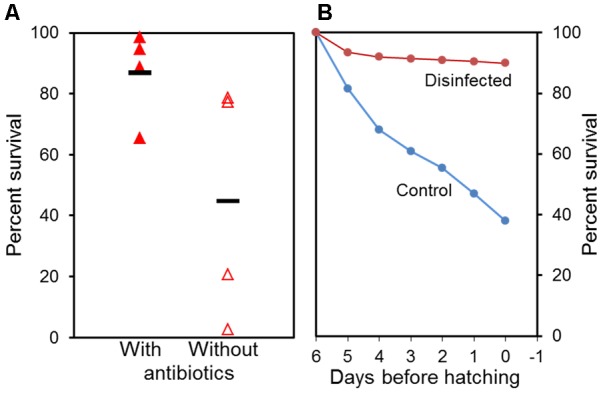
Effect of addition of antibiotics to yolk sac larvae **(A)** and surface disinfection of eggs **(B)** on survival of Atlantic halibut. **(A)** With and without 25 ppm oxytetracycline added to the water. Four independent replicates and average value are shown (horizontal line). **(B)** Disinfected, 400 mg glutaric dialdehyde per litre for 10 min; Control, without glutaric dialdehyde treatment. Data from Vadstein et al. (1993b).

Two examples involved use of surface disinfection of eggs and addition of antibiotics in the rearing of Atlantic halibut larvae (Figure 1). In both cases the survival was doubled when some type of control was taken over the microbes. Moreover, the variability between replicates was reduced about five times (Figure 1A).

In the late 1980s and early 1990s, only veterinarians were normally interested in microbes in aquaculture, and microbial problems in larviculture were considered small. Veterinarians were normally not able to identify specific pathogens that could explain the high mortality. This is an apparent contradiction with our claim above that harmful microbe–host relationships are a main cause of mortality in aquaculture. However, this apparent contradiction can be explained in three ways: (1) The pathogens of marine larvae were not yet identified and consequently veterinarians did not know what to look for; (2) The pathogens could not be cultivated by the methods used; and (3) The main reason for the observed problems were not true pathogens, but rather a dysbiosis of the normal microbiota due to opportunistic bacteria over-colonising a physiologically immature and stressed animal (Vadstein et al., 2004). These three explanations are not mutually exclusive, but we early hypothesised that the third explanation was the most likely for larval stages (Vadstein et al., 1993b) and below we will summarise evidence supporting this. For humans there are now more discussions of whether several diseases, e.g., persistent diarrhoea, are due to true pathogens or due to dysbiosis of the commensal microbiota (Sarker et al., 2017).

## An Analysis of the Problem and a Hypothesis for Dysbiosis: an Ecological Approach

Until the 1990s there were generally two approaches for microbiological studies in fish rearing; descriptive, cultivation-based studies listing which microbes were found (reviewed by Cahill, 1990), and studies of probiotics initiated by Joel Gatesoupe (Gatesoupe, 1991). Probiotics had already been tested in broiler production in the early 1970s (Nurmi and Rantala, 1973), and the introduction of probiotics represented a paradigm shift; from description aiming at understanding, to understand to be able to change. As a third approach, it was useful to view the first feeding environment with its microbes as an ecosystem that could be analysed using ecological theory, aiming at understanding the ecosystem with the intention of improving the conditions for the larvae (Vadstein et al., 1993b).

**FIGURE 2:**
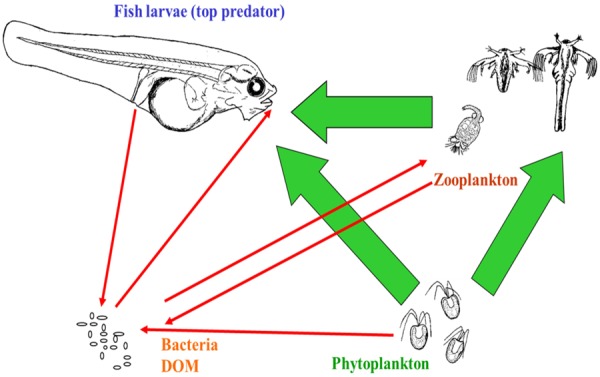
First feeding of larvae illustrated by a simplified food web. Arrows indicate main influences in the direction of the arrow. Interactions between dissolved organic matter (DOM) and bacteria, bacteria–bacteria interactions, and viruses are not indicated. In addition to interactions ongoing within the rearing tank, various inputs and losses from the tank also affect this microbe dominated food web. Figure from Vadstein et al. (2018).

Even a simplified outline of the first feeding food web (Figure 2) illustrates that a fish-rearing tank is a complex ecosystem. Although the focus in aquaculture is on the interaction between fish larvae and their live prey, there are at least seven additional interactions of importance in larval tanks to which microalgae are added. Live feed eat both algae and bacteria (e.g., Vadstein et al., 1993a; Makridis and Vadstein, 1999), and larvae actively take up bacteria and algae at rates that are typically 100 times higher than their drinking rates (Reitan et al., 1998). All organisms release dissolved organic matter (DOM), also the microalgae (Grossart and Simon, 2007). Also faces and uneaten food can be converted to DOM. In turn, DOM is a resource for heterotrophic bacteria, and the basis for their growth within the rearing system. The heterotrophic bacterial community in larval rearing systems is typically highly diverse and consists of hundreds of species (Bakke et al., 2015; Giatsis et al., 2015; Stephens et al., 2016). Consequently, the rearing food web with these four functional ecological groups (fish larvae, zooplankton as live feed, algae, and bacteria) probably contains thousands of biotic interactions. The inclusion of bacteria dramatically increases the complexity of the rearing system.

It is important to question which factors that regulate the number of microbes and the composition of the microbial community in the rearing system. The density of heterotrophic bacteria is determined by: (1) the supply of bacteria from all sources (water, live feed, and algae), (2) the growth of heterotrophic bacteria within the system based on the internal supply of DOM, and (3) the loss of microbes due to predation by live feed and larvae and virus infections. The second factor is related to the carrying capacity of the system, which determines the maximum sustainable bacterial biomass of the system.

The species composition of the bacterial community of the system is determined by (1) the composition of the bacteria in the external sources mentioned above, and (2) the growth potential and the selective forces acting on the microbial community within the system. The growth within the system (header tanks, tubes, and rearing tanks) can be substantial, and may contribute with 10^8^ bacteria L^-1^ h^-1^ (Attramadal et al., 2014; Vadstein et al., 2018). Depending on the rearing conditions, this is 1.2–14 times higher than the bacteria brought in with the incoming water. Consequently, the growth within the rearing system may strongly affect the species composition in the rearing tank. In the following, we will therefore focus on the supply of DOM, i.e., the growth substrate for heterotrophic bacteria, and selective forces in the rearing system (i.e., factors that select for certain species of bacteria). The aim of the analysis presented below is to understand which types of bacteria that are selected for during bacterial growth in the rearing system, and consequently which bacteria the larvae become exposed to.

Our understanding of the interactions between microbial species and the outcome of competition in microbial communities is inadequate and cannot be used for predictions of community composition (e.g., Larsen et al., 2012). This is also the case for bacterial interactions with fish larvae at the bacterial species level. An alternative approach is therefore needed. General ecological theory on selection and succession can be applied to predict how species are selected and how communities are formed in the fish rearing tanks. Here we will focus on the r/K selection and on the Pioneer-Mature community succession theories, and by this assign the bacteria into two functional groups, r- and K-selected bacteria, respectively. The r/K selection theory was proposed by Robert MacArthur and Edward O. Wilson in 1970 (Pianka, 1970). Even though the theory has been strongly criticised for not handling life history of animals in a good way, it seems to apply well to microorganisms that grow by binary fission and thus do not have a life history (Andrews and Harris, 1986). In the r/K selection theory, the selective pressure is hypothesised to drive selection in one of two generalised directions by favouring species with distinct growth strategies (Table 1). The r-selected species have high growth rates (r) at the expense of competitive ability and specialisation, and they rapidly colonise unexploited environments with high resource availability per bacterium. By contrast, K-selected species have traits that make them successful in crowded environments, with bacterial densities close to the carrying capacity (K), and they are competition specialists with high affinity for resources. Whereas K-strategists perform well at densities close to the carrying capacity, the r-strategists do not. The properties of r- and K-selected microbial species given in Table 1 can be used both for selection of desired species (more details below) and for diagnostic purposes (Salvesen et al., 1999; Salvesen and Vadstein, 2000). In fact, r-selected species are often referred to as opportunistic, and most pathogens are characterised as r-strategists (Andrews, 1984). In the context of aquaculture, the fast growth of a microbe is not a negative trait *per se*, but rather various traits associated to their opportunistic nature and the fact that most pathogens are r-strategists.

**Table 1:** Characteristics of r/K-strategic species and of pioneer/mature communities [slightly modified from a table in Vadstein et al. (1993b)].

Species level:	r-strategists	K-strategists
Maximum growth rate	High	Low
Biomass at carrying capacity	Unstable	Stable
Effect of enrichment/instability	Rapid growth	Slow growth
Competitive ability at low substrate supply per capita	Poor	Good
Affinity to substrate	Low	High

**Community level:**	**Pioneer**	**Mature**

Biological control	Low	High
Stability to perturbations	Poor	Good
Diversity (species, biochemical)	Low	High
Niche width	Wide	Narrow
Specialisation	Low	High

At the community level, r- and K-selected species dominate in pioneer (developmental) and mature (climax) communities, respectively (Odum, 1971). The distinct characteristics of these communities have consequences for community stability and resilience (Table 1). Pioneer communities are unstable systems with low stability against perturbations (low resilience) and with a low internal biological control. Matured systems inhabited by K-strategists have the opposite characteristics, and should consequently have more resistance against invasion by r-strategic microbes that are detrimental to the fish.

An ecological analysis indicates that current intensive production methods for rearing of marine fish larvae increase the carrying capacity of the system (more DOM) and tend to select for opportunistic microbes (r-strategists). This is due to the high and variable load of organic matter, which leads to perturbations of the bacterial community. The load of organic matter is directly related to the addition of feed, and indirectly due to defecation and excretion (Figure 2). The oscillations in the organic load are due to temporal fluctuations and to the difference in DOM between intake water and tank water. This is a main reason for r-selection in the rearing system (Table 1). Below, two types of perturbations are used to exemplify this.

**FIGURE 3:**
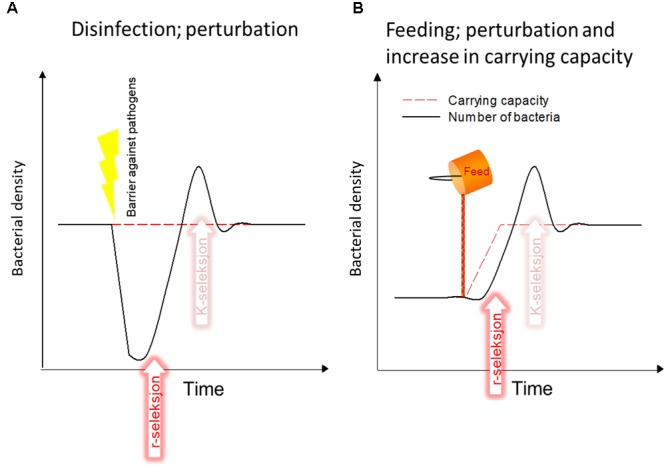
Two examples of how current aquaculture practises promote r-selection, increase the probability of opportunistic bacteria, and consequently the probability of detrimental larvae-microbe interactions. **(A)** Disinfection of intake water. **(B)** Addition of feed to the system. See text for details.

The first example of a perturbation that creates r-selection in larval rearing is disinfection (Figure 3A). Disinfection of intake water is used as a necessary barrier against introduction of pathogens in the system. However, disinfection also decreases bacterial numbers, it results in more DOM released from dead organisms, and it makes DOM more bioavailable (Hess-Erga et al., 2010). This will potentially result in a subsequent bloom of fast-growing r-strategists, unless the retention time in the tanks is very short. The concentration of bacteria will be more or less the same as before the disinfection because the carrying capacity is not changed.

The second example of a perturbation is addition of feed, which is live feed and algae in the early larval phase, and formulated feed in later stages (Figure 3B). The addition of feed increases the availability of DOM directly, but also indirectly through defecation/excretion by all organisms in the system, i.e., fish, live feed, and algae. In contrast to disinfection, addition of feed will significantly increase the microbial carrying capacity in the rearing tanks. This will also most likely result in r-selection. It is common in larval rearing that the water typically resides in the rearing tanks during the period with r-selection (just after disinfection and feeding), and has already left the tanks when a potential K-selection can take place. This will normally be different for later stages with fast exchange rates of tank water.

Based on the above ecological analysis, we have proposed the following hypothesis: Traditional rearing methods result in r-selection for opportunistic microbes, and this increases the probability for detrimental fish–microbe interactions. This hypothesis involves a cascade of events, and a proper test of the hypothesis requires verification of effects of the selection regime at three linked levels. First, the effect of the rearing method/technology on the microbial community composition of the water. Second, the effect of the microbial community composition of the water on the composition of the microbiota of the larvae. Third, the effect of the composition of the microbiota of the larvae on their overall performance (viability). There are many examples of studies on which data on the microbiota of fish are inadequate for evaluating this cascade. The predicted consequences of r-selection on microbial communities are: reduced diversity, dominance by fast-growing opportunists, low stability and biological control within the microbial communities, and increased susceptibility to invasive species (Table 1). First, we will provide data for testing our hypothesis on r-selection for the composition of the water microbiota.

**FIGURE 4:**
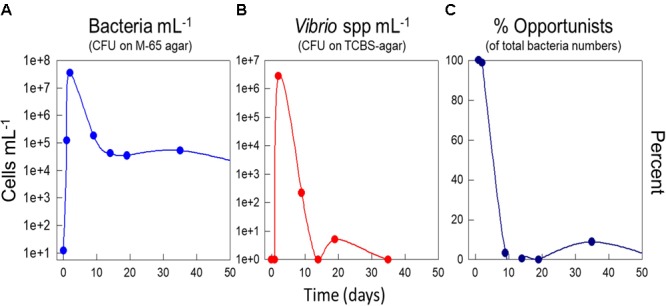
Effect of a perturbation by disinfection and a shift-up in DOM on day zero by addition of yeast extract. Effect on total counts of culturable bacteria on M65 agar **(A)**, counts of presumptive *Vibrio* spp. based on plating on TCBS agar **(B)**, and percentage of opportunistic bacteria estimated as the fraction of fast-growing colonies on M65 agar **(C)**. Experiments in seawater at 15°C. Data from Salvesen (1999).

Early work using cultivation dependent methods (Salvesen, 1999) showed that disinfection and pulsing with nutrients resulted in a bloom of bacteria, and that this bloom was totally dominated by presumptive *Vibrio* species and opportunistic r-strategists (Figure 4). Moreover, we also showed that the percentage of culturable bacteria was higher under r-selection than under K-selection, which is an indicator of less specialists, and hence r-strategists, in the system (Salvesen et al., 1999).

**FIGURE 5:**
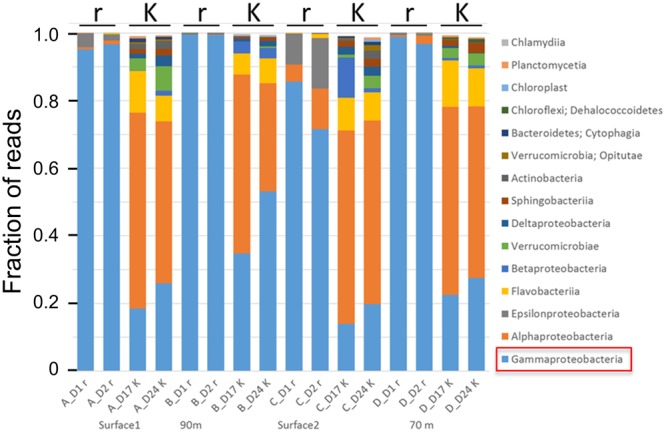
Effects of r- and K-selection on microbial community composition in four different experiments with different seawater inoculum. Each experimental group was sampled twice during the periods of r- and K-selection. r-selection was obtained by pulsing with resources for growth (organic and mineral nutrients), whereas K-selection was obtained by resource depletion and competition for more than 2 weeks. r-selected communities were sampled at days 1 and 2 after addition of resources, whereas K-selected communities were sampled on days 17 and 24. Microbial community composition was analysed by Illumina sequencing of 16S-rDNA amplicons. Unpublished results.

A later study using culture independent Illumina sequencing of 16S rRNA amplicons of bacteria supported these findings and provided some additional information (Figure 5). In four batch reactors with different natural seawater samples as inoculum, we initiated r-selection by addition of DOM resources for growth of bacteria on day 0 of the experiments, and K-selection was obtained by the competition taking place after consumption of the initial pulse of resources. For all four experiments the community composition at the phylum level was distinctly different in the r- and K-selection phases. Whereas r-selected communities were dominated by γ-proteobacteria (typically >80%), the K-selected communities were more diverse comprising α-proteobacteria, Flavobacteria, β- and δ-proteobacteria, and Verrucomicrobia as the most abundant phyla. The diversity number (defined as e^Shannon^; Jost, 2006)) was six times higher in the K-selected than in the r-selected communities (57.8 ± 23.1 versus 9.1 ± 4.2), and the operational taxonomic unit (OTU) richness was three times higher (550 versus 180 OTUs). The γ-proteobacteria that dominated in the r-selected communities included many genera known to contain pathogens (e.g., *Vibrio, Pseudomonas, Aeromonas, Pseudoalteromonas, Yersinia, Shewanella, Legionella*, and *Enterobacter*), supporting the link between r-strategists and pathogens. A similar increase in the abundance of γ-proteobacteria has been found after water disinfection and subsequent r-selection by Becerra-Castro et al. (2016).

We can conclude that different microbial community composition is created under r- and K-selection, and that simulations of the perturbations that are common in larval rearing creates r-selection. The above experiments did not provide evidence for the implications of r-selection on the microbiota of larvae or on the viability of larvae. For a proper test of the hypothesis on r-selection and dysbiosis with respect to larval microbiota and viability, we need to compare traditional rearing technology creating r-selection to alternative technologies creating K-selection. This is the focus below.

## A Hypothesis for Microbial Community Management and Two Methods for K-Selection

The hypothesis above on r-selection and dysbiosis of larvae point to a solution to the current problems in larval rearing, and it serves as a basis for proposing a microbial community management hypothesis: K-selection in the rearing environment will select against detrimental r-strategic microbes and thus promote healthy microbe-larvae interactions. This has been a core hypothesis for our work for more than 25 years, and we have used this hypothesis to develop a strategy for microbial control in larval rearing.

It is practically possible to create K-selection in an aquaculture system that is open for all sorts of microbes. K-selection can be set up by securing persistent competition, i.e., low resource supply per bacterium (Table 1). This can be achieved by maintaining a high bacterial density and/or by reducing resource supply. Competition between bacteria is not a matter about substrate concentration *per se*, but rather the supply per bacterium per unit of time. The degree of competition will therefore depend on both the rate of resource supply and the number of bacteria present in the system.

It is also important to consider that in aquaculture systems the resource supply should not be reduced to a level considerably lower than the carrying capacity in the rearing tanks (we will come back to this later. See Prediction 2). Consequently, to obtain K-selection in aquaculture systems by means of strong resource competition, we suggest to increase the number of bacteria present in situations where resource availability allows substantial bacterial growth. We have previously proposed two methods to secure K-selection, both of them based on increasing the number of bacteria competing for the limiting DOM resources in the system.

### Microbially Matured Water System (MMS)

This method is a modification of a classical flow-through system, where a hygienic barrier obtained by disinfection of intake water with UV/ozone or ultra-filtration is the first step. Whereas bacterial recolonisation normally starts in the header-tank, the recolonisation for MMS takes place in a header-tank with a biofilter. After some time of conditioning, the biofilter has a considerable biomass of attached bacteria that is scaled to the supply of DOM, and efficient competition and K-selection is thus obtained. Some of the bacteria in the biofilm will detach and supply the bacterial community of the water with K-selected biofilm bacteria. The dynamics of the competition between pelagic and attached bacteria, the significance and kinetics of the detachment of biofilm bacteria for the microbial community composition of the water, and the role of the water retention time in the header-tank on the degree of K-selection is not fully understood. The MMS was proposed and tested for the first time by Vadstein et al. (1993b).

### Recirculating Aquaculture System (RAS)

These are systems with high reuse of water (>90%) that traditionally have been designed with removal of particles by mechanical processes and detoxification of ammonia by biological processes (nitrification by chemoautotrophic bacteria) in a biofilter (Summerfelt and Penne, 2005; Martins et al., 2010). However, most of the bacteria in the biofilter are heterotrophic and rely on the supply of DOM (Michaud et al., 2006; Blancheton et al., 2013; Gonzalez-Silva et al., 2016). As most of the DOM is consumed in the biofilter, these heterotrophic bacteria may, as in MMS, influence the microbiota of the water (Leonard et al., 2002). The number of heterotrophic bacteria in the biofilter will increase until there is a balance between total cell number, DOM supply/growth, and loss rates from the biofilm (Rojas-Tirado et al., 2017). Under these conditions there will be competition for resources, hence leading to K-selection.

From their early development, the biofilters in RAS have empirically been dimensioned to support the growth of both heterotrophic bacteria and nitrifiers. This is not because heterotrophic bacteria have been of particular interest, but because they are better competitors for oxygen due to good growth at low concentrations. Heterotrophic bacteria will outcompete nitrifiers for limiting resources such as space and oxygen, if these resources are not provided in adequate amounts. However, RAS *per se* does not secure K-selection because it depends on the overall design of the system. For example, disinfection of water within RAS may compromise K-selection (see Prediction 3 below). The long retention time of water in the system will, however, strengthen K-selection. The conception of RAS as systems for K-selection is new, and it was tested for the first time by Attramadal et al. (2012a), although proposed by Salvesen (1999) in her Ph.D. thesis.

In the following sections, we will present the results from testing our hypothesis for microbial management by K-selection and three predictions based on this hypothesis (Table 2). In most cases this has been done in a rigorous, mechanistic way by including testing of the consequences of K-selection in the cascade from water microbiota to fish microbiota and finally to the viability of larvae (Table 2).

**Table 2:** Overview of our microbial management hypothesis and predictions from this hypothesis discussed here.

**Hypothesis:**	K-selection in the rearing environment will select against detrimental r-strategic microbes and thus promote healthy microbe-larvae interactions
**Predictions (P):**	
P1	K-selection in in-flowing water at a carrying capacity similar to the one in rearing tanks will result in less r-selection in the rearing tanks
P2	RAS is a method for K-selection of microbial communities that improves the viability of fish larvae
P3	UV treatment before rearing thanks causes r-selection in tanks and detrimental microbe-larvae interactions

*A proper testing of hypothesis and predictions requires verification of a cascade of events from the effects of K-selection on the microbiota of water, to the microbiota of larvae, and finally to the viability of larvae.*

*The hypothesis on r-selection and dysbiosis of larvae is not included in this table (see text)*

## Hypothesis: K-Selection in the Rearing Environment Will Select Against Detrimental R-Strategic Microbes and Thus Promote Healthy Microbe-Larvae Interactions

First, we will present experimental data to test if K-selection entails changes in the composition of the water microbiota. Some early studies, give support to the dominance of K-selected microbes in MMS relative to conventional flow-through systems (FTS). This was observed in experiments with Atlantic halibut and turbot larvae and use of culture-dependent microbiological methods and total counts of bacteria by epi-fluorescence microscopy (Vadstein et al., 1993b; Skjermo et al., 1997; Salvesen et al., 1999). A low percentage of fast growing bacteria and a low percentage of culturable bacteria were early proposed as indicators of mature microbial communities dominated by K-strategists (Skjermo et al., 1997; Salvesen and Vadstein, 2000). Contrarily, high fractions of fast-growing opportunistic and culturable bacteria were supposed to reflect a dominance of r-selected bacteria.

It has generally been found that on agar plates with so-called non-selective medium, the measured percentage of fast-growing bacteria (those visible on plates after 3 versus 21 days of incubation) was normally higher in FTS than in MMS tank water, indicating more bacteria with high maximum growth rates (i.e., r-strategist) in FTS than in MMS (Skjermo et al., 1997; Salvesen et al., 1999). Moreover, the percentage of culturable bacteria relative to total counts was considerably lower in MMS than in FTS tank water, indicating more specialists (i.e., K-strategists) unable to grow on agar plates in MMS than in FTS.

**FIGURE 6:**
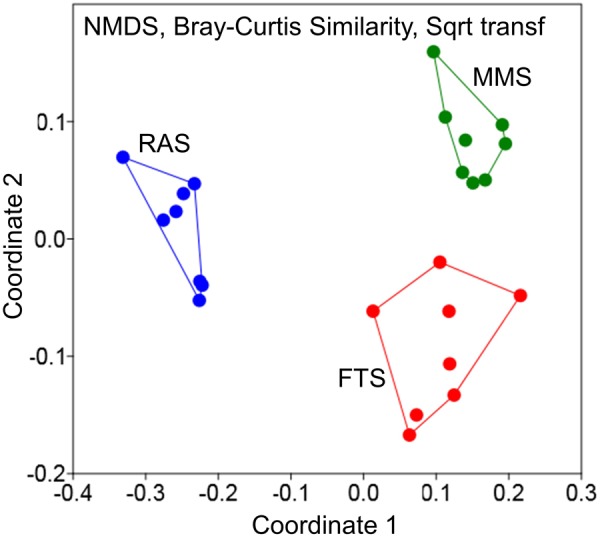
Differences in microbial community composition of inflowing water in two assumed K-selected (MMS and RAS) and one r-selected (FTS) larval rearing systems (data from Attramadal et al., 2014). The water was sampled nine times during 1 month, and each point represents one sample.

A recent study used a cultivation independent DNA fingerprinting method (analysis of 16S rDNA amplicons by Denaturing Gradient Gel Electrophoresis; PCR/DGGE) to compare the composition of the bacterial communities of inflowing and tank water in one r-selection (FTS) and two K-selection systems (MMS and RAS) in a first feeding experiment with Atlantic cod larvae (Attramadal et al., 2014). The inflowing water was sampled nine times during 1 month. Ordination based on Bray–Curtis similarity showed a clear separation of the microbial community composition of the three water treatment systems (Figure 6), and statistical analysis confirmed it (PERMANOVA, *p* < 0.0001). Moreover, as indicated in Figure 6, the two K-selection systems created microbial communities in the in-flowing water that were more stable over time, and “moving window” analysis confirmed that the average Bray–Curtis similarity between bacterial communities over time was significantly higher in the K-selection treatments (MMS and RAS) than in the FTS (*p* < 0.001; Attramadal et al., 2014). Various alpha-diversity indices calculated from the DGGE-data, showed clear tendencies for higher richness and evenness in MMS and RAS water than in FTS. When the composition of the microbial community was compared for in-flowing and tank water, the Bray–Curtis similarity was typically 75% in RAS and 20–25% in the other two systems (Attramadal et al., 2014).

In conclusion, with reference to the hypothesis above, microbial community management by K-selection using either MMS or RAS technology is working for in-flowing and tank water. Microbial communities associated with RAS and MMS had higher richness and evenness compared to those of FTS, and they had a lower fraction of fast-growing opportunistic bacteria. Moreover, microbial communities in K-selected systems (RAS and MMS) were more stable over time than communities under r-selection (FTS). All these observations are consistent with the r/K and Pioneer/Mature theories, and support our microbial management hypothesis with respect to the effects of K-selection on the microbial community characteristics of the water.

Further verification of part two of our above hypothesis requires documentation of whether r-selected pioneer (FTS) and K-selected mature (MMS and RAS) microbial communities of the water have implications for the composition of the fish microbiota. The first tests of our hypothesis were performed using MMS technology, before cultivation independent methods where generally available (Vadstein et al., 1993b; Skjermo et al., 1997; Salvesen et al., 1999). Accordingly, there is no data from these studies on the effects on the microbial community of the larvae. However, such data are available from later experiments, and are presented in detail below. In summary, all the experiments showed that changes in the microbiota of the water induced significant changes in the microbiota of the larvae. There are some additional important conclusions from these studies. First, the microbiota of the water affected the microbiota of the larvae more strongly than the microbiota of the live feed did (Bakke et al., 2013, 2015), emphasising the importance of water treatment. Second, there seems to be a strong selection in the host, and the microbiota of the larvae was not a mirror image of the water microbiota (Bakke et al., 2015). Finally, steering of the microbiota of larvae require a continuous treatment, as the effect of management is not lasting (see Prediction 2 below). This is probably because the microbiota of larvae changes continuously with larval development, most likely due to a concomitant change in the selection pressure (Bakke et al., 2015).

**FIGURE 7:**
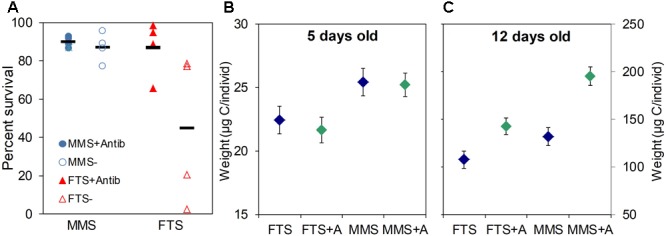
Comparison of microbially matured water (MMS) and water from a flow-through system (FTS) on larval viability. Survival of Atlantic halibut yolk sac larvae (day 43 after hatching) reared in both water types with (closed symbols) and without (open symbols) addition of 25 ppm of the antibiotic oxytetracycline **(A)**, growth of turbot larvae reared in both water types with (green symbols) and without (blue symbols) addition of microalgae for 5 **(B)** and 12 **(C)** day old larvae. Details: **(A)** Horizontal markers represent average values; Data from Vadstein et al. (1993b). **(B,C)** Average size (±SE) of turbot larvae; Data from Salvesen et al. (1999).

The effect of K-selection by MMS technology on the survival and growth of larvae was very clear in all experiments, including the data shown in Figure 7. The survival of yolk sac larvae of Atlantic halibut was doubled in MMS compared to FTS, and the survival in MMS without antibiotics added to the water was similar to the survival in FTS with antibiotics (Figure 7A). Moreover, reproducibility was improved more than eight times (CV of MMS < 0.1, versus FTS = 0.80). In a factorial design first feeding experiment with turbot using water treatment (MMS versus FTS) and addition of algae (with/without) as the two factors (Figures 7B,C), the weight of larvae reared in MMS was 15% higher than that of larvae reared in FTS on day 5 after hatching, and there was no effect of adding algae at this stage. On day 12, the weight of larvae was 22 and 37% higher for conditions without and with algae, respectively. Moreover, on day 19 there was a 100% difference in the average size of larvae between the two extreme groups (FTS without algae and MMS with algae; Salvesen et al., 1999). Thus, rearing of larvae in K-selected versus r-selected microbial communities resulted in earlier onset of growth. Due to the exponential growth of larvae, a small difference will materialise into large differences with time. It is not known to what extent the effect of the algae was related to the microbial conditions, but this is one of the mechanisms proposed for the positive effect of adding algae to the water (Reitan et al., 1997). Two independent experiments with turbot showed similar results, but with even larger effects on larval growth (Skjermo et al., 1997).

We can conclude that our hypothesis on K-selection for microbial management is supported rigorously by data on microbial community characteristics of water and larvae and viability of fish larvae. The data presented above are from two very different types and independently replicated experiments, and this provides robustness to our findings. Moreover, the data presented below, to test predictions from our hypothesis, provide additional support (see Table 2).

## Prediction 1: K-Selection in In-Flowing Water at a Carrying Capacity Similar to the One in Rearing Tanks Will Result in Less R-Selection in the Rearing Tanks

In MMS, the incoming water is matured in a header tank at a carrying capacity lower than the carrying capacity in the rearing tanks, as rearing tanks have higher loading of DOM due to feeding. Consequently, opportunists might proliferate in the tanks, depending on the hydraulic retention time (Figure 3B). If the carrying capacity in the MMS maturation unit is increased by continuous addition of DOM, this gap in carrying capacity between in-coming and tank water becomes smaller and there is thus less room for proliferation of r-strategists in the rearing tanks (Prediction 1; Table 2).

In a first feeding experiment, Ballan wrasse was reared in two MMSs operated at different carrying capacities. In one treatment, the F-MMS, the carrying capacity for heterotrophic bacteria was increased by feeding the biofilter with mashed fish feed as a source of DOM for the heterotrophic bacteria. In the control treatment, no fish feed was added to the MMS (Attramadal et al., 2016).

The water leaving the maturation units contained on average 8 × 10^6^ and 4 × 10^5^ bacteria mL^-1^ (total counts) in F-MMS and MMS, respectively. This confirmed the predicted increase in the microbial carrying capacity in the F-MMS. More important, the average number (±SE) of bacteria in the rearing water was 86 ± 13% and 245 ± 33% of the number of bacteria in the incoming water to F-MMS and MMS, respectively. Thus, the net microbial growth potential in tank water was not significantly different from zero and more stable in F-MMS than in MMS with substantial and variable microbial growth in the tanks. This substantial bloom of bacteria in the tank water of MMS resulted in selection for r-strategists under the prevailing conditions (defined above). In the F-MMS this was avoided by the increase in the carrying capacity of the maturation unit.

**FIGURE 8:**
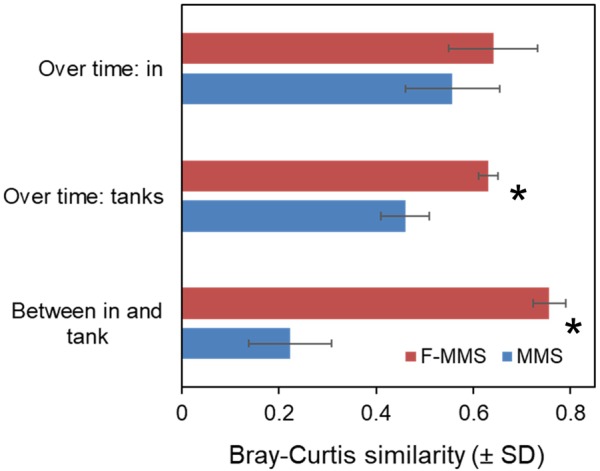
Effects of increasing the carrying capacity of incoming water on microbial community composition of in-flowing (in) and tank water, illustrated by a comparison of a normal MMS (Microbially Matured System) and F-MMS (MMS with mashed fish feed added in the maturation unit). Similarities in community composition were calculated as Bray–Curtis similarity from PCR-DGGE data. Significant differences between MMS and F-MMS are indicated by ^∗^. Data from Attramadal et al. (2016).

The microbial community compositions of the in-coming and tank waters in the Ballan wrasse experiment were analysed by PCR-DGGE. The stability of the microbial community compositions over time was 37% higher in the rearing tanks of the F-MMS than in the MMS (*p* = 0.004). As predicted, the stability of the microbial communities of the incoming water was not significantly different between the F-MMS and MMS (*p* = 0.334). The fact that the Bray–Curtis similarity between microbial communities in in-coming and tank waters was typically 0.76 in fed-MMS and only 0.22 in MMS (Figure 8) strongly supports our prediction. There were also significant differences in the microbial community compositions of MMS and F-MMS, and similarity in the microbiota between replicate tanks was typically 40% higher for F-MMS than for MMS. Moreover, the similarity in the composition of the microbiota of individual fish was typically 40% higher for F-MMS than for MMS (Attramadal et al., 2016).

In conclusion, this study demonstrated that it is possible to increase the carrying capacity in a MMS by adding DOM to the biofilter. This stabilises the microbial community in time and between replicate tanks, and prevents proliferation of r-strategic bacteria in the rearing tanks. The experiment supports our Prediction 1 above that K-selection in in-flowing water at a carrying capacity similar to the one in rearing tanks will result in less r-selection in the rearing tanks.

Recirculating aquaculture system represents another strategy to mature the microbiota at a carrying capacity similar to the one in the rearing tanks (Attramadal et al., 2014). Also this experiment provided support to Prediction 1 and showed that RAS will generate K-selection of the microbial community at a carrying capacity similar to the one in the rearing tanks, and thus limit the possibility for growth of bacteria and r-selection in the rearing tanks. Details are given under Prediction 2.

## Prediction 2: Ras Is a Method for K-Selection of Microbial Communities That Improves Viability of Fish Larvae

It is a well-established fact that microbes are fundamental to the function of RAS, but this has mainly been attributed to the nitrifying bacteria that detoxify ammonium by converting it to nitrate (Blancheton et al., 2013). The reasoning for and the advantages of using RAS as a K-selection method were given above, but data to support them has so far only been given for water microbiota, i.e., RAS selects the microbial community of the water consistent with RAS functioning as a K-selection method for microbial community management. To test Prediction 2 properly, we also need data on microbiota and viability of larvae. Moreover, it is interesting to compare the two K-selection methods we have proposed (i.e., MMS and RAS) as we have stated that these selection systems are different with respect to carrying capacity and total system hydraulic retention time. These differences can produce significant effects both in the microbiota and in the viability of larvae, which is partly related to Prediction 1.

**FIGURE 9:**
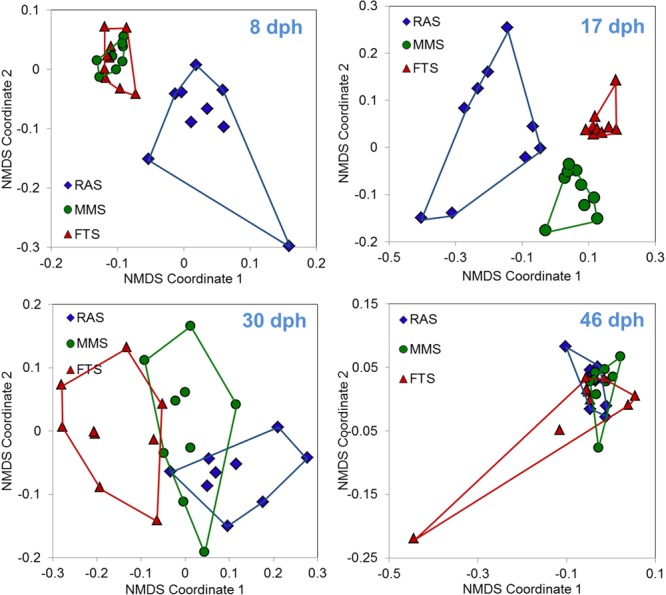
Ordination based on non-metric multi-dimensional scaling using Bray–Curtis similarities of microbiota associated with cod larvae reared in FTS, MMS, or RAS. Points are for individual fish from three replicated tanks sampled at four different days post hatching (dph). Data are based on PCR-DGGE. From dph 31 onwards all tanks received the same water type (MMS); thus, data from 46 dph represent fish with identical water for 2 weeks, but with different original microbial environment. Data from Truong (2012).

Samples from a first feeding experiment with Atlantic cod comparing FTS, MMS, and RAS (Attramadal et al., 2014) have been used to characterise the microbiota of larvae by PCR-DGGE (Truong, 2012). These data support our Prediction 2 on the effect of RAS as K-selection systems on the microbiota of larvae (Figure 9). The fish microbiota at 17 and 30 days post hatching (dph) showed that there was a clear grouping of samples related to the system used for water treatment, and significant differences in the composition of the microbiota of the larvae among the three rearing systems (PERMANOVA, *p* < 0.006). At 8 dph, only RAS was separated from the other two systems. All tanks received that same water (MMS) from dph 31 onwards, and this resulted in no grouping of larval microbiota according to initial water treatment method 2 weeks later, and no statistically significant differences in bacterial community composition among groups (*p* = 0.19 at 46 dph). Although we only have data on the microbiota of larvae from one experiment, these strongly support the prediction that RAS as a K-selection method (Prediction 2) affects the microbiota of the water in a way that results in changes in the microbiota of larvae. Moreover, we have indications that RAS, compared to MMS, has the ability to influence the microbiota of larvae at an earlier stage (Figure 9). It is possible to reduce the carrying capacity in RAS by reducing DOM input using clay instead of algae in the water (Attramadal et al., 2012c) or using ultrafiltration to remove DOM (Wold et al., 2014).

**FIGURE 10:**
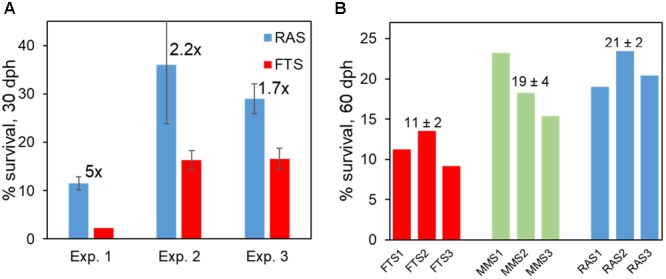
Survival of Atlantic cod larvae in RAS and FTS **(A)**, and in both K-selection methods (MMS and RAS) and r-selection (FTS) **(B)**. Numbers in **(A)** close to the RAS bar indicate effect size (RAS/FTS), and numbers on top of bars in **(B)** are average ± SD. Data from Attramadal et al. (2012a,b, 2014).

In several RAS experiments, we also found support for an improved viability of cod larvae. This included earlier onset of growth of larvae, faster growth of larvae (Attramadal et al., 2012a,b), and at least 70% increase in survival at 30 dph in RAS (Figure 10A). It is interesting to note that in one experiment both K-selection systems resulted in 65–70% increase in survival (Figure 10B).

In conclusion, our Prediction 2 on RAS as a K-selection method is supported by data on water and larval microbiota and on larval viability. The quantitative effects on the viability of larvae are pronounced when compared with FTS (e.g., 70% increase in survival). Moreover, whereas both K-selection methods had comparable effects on the viability of larvae, RAS provided higher stability in the microbial community composition over time and less r-selection in the rearing tanks. This is because RAS do not have a large gap in carrying capacity of bacteria between in-coming and tank water, and that the hydraulic retention time in RAS is high and results in a prolonged selection period. We have not tested the fed MMS (F-MMS, Prediction 1) versus RAS, and cannot therefore conclude that RAS is a generally better K-selection method than MMS.

## Prediction 3: Uv Treatment Before Rearing Thanks Causes R-Selection in Tanks and Detrimental Microbe-Larvae Interactions

Disinfection of the intake water is commonly used for biosecurity in aquaculture. In RAS, disinfection is also often used within the treatment loop, but there is no overall agreement for where in the loop it should be placed. Some people argue to place UV-treatment before the rearing tanks to provide low microbial load for the reared organisms, and some argue to place it before the biofilter as a hygienic barrier. Based on our hypothesis, we predict that disinfection of the water just before entering the rearing tanks will cause r-selection in the rearing tank. The large difference in bacterial density in the water after disinfection compared to the carrying capacity for bacteria in the rearing tank, will cause r-selection and undesirable changes in the composition of the microbes in the water, with negative consequences for the viability of the larvae.

We tested Prediction 3 in two experiments with European lobster larvae, using RAS without and with UV treatment of the in-flowing water (RAS and RAS-UV) (Øien, 2014; Attramadal et al., unpublished). We compared these two RAS configurations with a conventional FTS, which would promote r-selection of heterotrophic bacteria in the tanks (see above). We hypothesised that the r-selection in FTS will be less pronounced than in RAS-UV because the bacterial density in the in-flowing water is higher in FTS.

**FIGURE 11:**
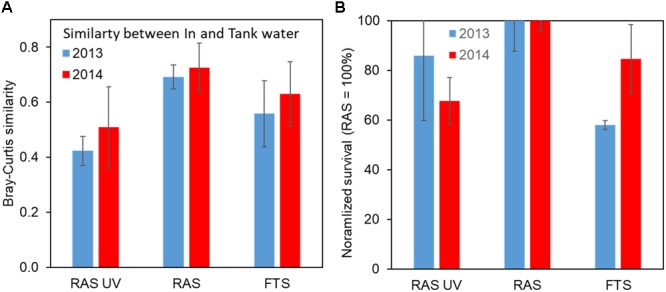
Similarity in bacterial community composition in in-flowing and tank water **(A)** and normalised survival of lobster larvae (**B**; RAS = 100%) for the different treatments. FTS, Flow through system; RAS, Recirculating aquaculture system; and RAS-UV, RAS with UV treatment of in-flowing water. Error bars are standard error for six comparisons **(A)** and duplicates **(B)**. Data from Øien (2014) and Attramadal et al. (unpublished).

The UV treatment clearly affected the composition of the bacterial community in the rearing tanks, with a 37% reduction in the similarity with the in-flowing water community before disinfection (Figure 11A). As hypothesised, the degree of change in the bacterial community composition between in-flowing water and rearing water in FTS was intermediate compared to the two RAS configurations. The different rearing systems also influenced the microbiota of the larvae. Significant differences were found in the microbiota of the lobster larvae between FTS and RAS, and between FTS and RAS-UV for all sampled days (4, 8, and 12 dph, *p* < 0.05), and on 12 dph between RAS and RAS-UV (*p* = 0.048) (Øien, 2014).

In both experiments the survival of larvae until stage IV (14 and 21 dph for Experiments 1 and 2, respectively) was reduced by on average 23% as a result of the UV-treatment (Figure 11B). Moreover, as hypothesised the rearing of lobster in FTS showed less predictable survival, with larger year-to-year variations. The reduced survival in the RAS-UV treatment was not statistically significant (*p* = 0.146) due to low power (only two replicates).

In a separate study with rearing of Atlantic cod larvae, we observed a 40% reduction in survival at day 30 after hatching when UV-disinfection was used in RAS, and the survival in RAS-UV was similar to the survival in FTS (Attramadal et al., 2012b). The UV-treatment also destabilised the microbial community in the rearing tanks. Thus, also these data support Prediction 3 regarding the negative effects of UV treatment before the rearing tanks, by documenting effects on the microbiota of the water and on viability of larvae.

In conclusion, both experiments provided support for Prediction 3; the UV-treatment affected the microbiota in the rearing tanks, that affected the microbiota of larvae, affecting finally the survival of both lobster and cod larvae. This suggest that UV-treatment just before the rearing tanks should be avoided. One modifying factor for this conclusion is the dilution rate/retention time of the water in the rearing tanks. We have data from smolt production of Atlantic salmon showing no change in the microbiota of the tank water in a system with UV treatment of in-flowing water (unpublished results). In this case, the hydraulic retention time was so short (30–45 min) that the bacteria did not have time to divide before leaving the tank with the water (Bakke et al., 2017), and most of the DOM was likely consumed in the biofilter.

## Is K-Selection as a Microbial Community Management Strategy Relevant at Industrial Scale?

Several persons working in the aquaculture industry and consultants for the hatchery industry that we have spoken to, have confirmed that, the microbial environment is a major problem in commercial hatcheries. Whether this is considered the major problem or one of the major problems varies, but most agree that microbial problems are more complicated because proper diagnostic tools and protocols for counteraction are not available.

Few relevant experiments have been performed at industrial scale that can be used for evaluating our hypothesis and the predictions discussed above. Two facilities for larval rearing of Atlantic halibut have tested a fed MMS and an internal RAS with a biofilter on the side of each tank. Although the design of these experiments had some limitations, they both indicated substantial improvement in survival by changing to a K-selection technology. With internal RAS, the survival was improved by a factor of 10 and 6 in two following years, and the facility has now changed their technology to internal RAS, and they consider that K-selection is important for success in larval rearing. Fed MMS has also improved production results according to the fish farmer, but it has not been compared directly with FTS for the same group of fish at industrial scale.

It is also possible to evaluate whether the hypothesis on microbial management by K-selection and predictions derived from the hypothesis are consistent with observations on industrial scale or not. Several respected scientists with a close connexion to the industry confirm that the r/K-selection hypothesis is consistent with their observations (Patrick Sorgeloos, Jean Paul Blancheton, and Joana Sapo pers comm). These observations cover a large number of species and geographical areas. It is interesting that many opinions on microbial management are related to RAS – an area in which hardly any relevant research has been done.

It is reasonable to conclude that the microbial problems documented at laboratory scale and countermeasures by K-selection are relevant also at industrial scale. The few experiments and observations made at industrial scale suggest that attempts to improve microbial conditions according to the principals documented above may substantially improve performance of larvae and quality of juveniles.

## Impact of K-Selection on Microbiota and Larval Viability: General Discussion and Conclusions

This review, mainly based on previously published results, strongly supports the hypothesis that r-selection in the microbial community is a main reason for the problems/crashes observed in larviculture in the past and nowadays. More important, these problems might be counteracted based on the use of the hypothesis on microbial management by K-selection and the three predictions derived from this hypothesis (Table 2). All experiments presented here support our hypothesis and the predictions made from this hypothesis. The robustness of our hypothesis is indicated by the fact that experiments have been performed with five different species, including both fish and crustaceans, by experiments in both MMS flow-through systems and RAS, and conducted during 25 years.

Our analysis and experimental data point to microbial management strategies focusing on bacterial community composition rather than to bacterial densities as a key factor for success in larviculture. We advocate that this conclusion is generally applicable also for other farmed animals like chicken and piglets, where mortality is a problem for young stages. An advantage with our approach is that we select for organisms with an ecological growth strategy to avoid detrimental microorganisms. We assume that by promoting K-selection we obtain low abundance of detrimental microbes and high diversity of K-strategists from which the larvae can select a healthy microbiota. Such an indirect strategy is in fact required, as species lists of microbes needed to obtain a functional host microbiota do not exist in neither natural nor domesticated situations.

Although the technologies for obtaining K-selection are already applicable at industry scale, some knowledge gaps still exist, especially for the MMS approach. First, we should know more about the types of species (OTUs) selected for in both K-selection systems (i.e., MMS and RAS). The limited data existing is mainly from conditions of extreme r- and K-selection, and we do not know whether γ-proteobacteria dominance under r-selection holds in aquaculture rearing systems with more moderate r-selection pressure. Second, we should investigate how resistant these systems are to invasion by harmful microbes. This is particularly relevant for RAS, as an emerging problem can accelerate due to the recirculation of water and microbes. Third, we need more knowledge on design criteria for K-selection systems at industry scale. For MMS this includes more knowledge on their capacity to produce stable mature microbial communities, and for RAS the configuration/order of system components and the regulation of carrying capacity. For MMS, the main focus should be on the capacity of the header-tank with the biofilter to produce mature microbial communities as a function of hydraulic retention time and surface area per volume. For both MMS and RAS we need a better understanding of the characteristics and relationships between free-living and surface attached microorganism in the system. Finally, more knowledge should be established on the importance of water microbial communities for microbial community assembly in developing larvae, and on the functional role of the microbiota for healthy development of larvae. As a consequence, we claim that there should be a shift in focus with more attention on the microbiota of healthy fish relative to the attention on pathogens and sick fish.

It is important to evaluate the feasibility and the magnitude of effects on larvae of methods for microbial community management by K-selection relative to other microbial management methods. Methods focusing on the density of microbes have so far received most attention (e.g., disinfection, filtration and cleaning). As part of normal hygiene routines, these methods are important. However, as we have shown, they may result in perturbations promoting r-selection and an unfavourable microbial environment. The methods aiming at reducing the density of microbes may therefore create more problems than they solve, if the r-selection they promote is not properly mitigated.

For controlling the microbes that are present, we are only aware of one other proposed method. That is addition of health promoting bacteria to the system – the probiotics concepts. Probiotics have been studied in aquaculture for about 30 years. There are several positive reports in the literature and a large number of reviews have been published, most concluding that the use of probiotics has positive effects. We suggest that K-selection will work better, especially when it comes to the effects on the larvae. Moreover, from a theoretical point of view, we argue that there are some inherent problems with the use of probiotics in larviculture that have not been properly studied. First, the addition of probiotics is a dramatic perturbation of the microbial community, which will reduce microbial diversity and may entail a shift to higher abundance of r-strategists and less invasion resistance toward pathogens (De Schryver and Vadstein, 2014). Second, e.g., Skjermo et al. (2015) indicated that probiotic management of the microbial community of larvae lasts for a limited period – i.e., a few days. This can also be predicted from principles in microbial ecology as a developing larva is an ecosystem with a continuous change in selection pressure (Bakke et al., 2015). This knowledge is in contrast to most probiotic studies that involve a single and likely short-lasting perturbation of the microbial community. Finally, it should also be noted that probiotics have lately received less attention for humans after the successful introduction of gut microbiota transplantation to treat problematic infections like *Clostridium difficile* (see e.g., Brandt, 2013). It is interesting to note that this new approach, similar to K-selection, is a microbial community management approach and does not use one or a few species of microbes.

We hope that this review has demonstrated the power of basic microbial ecology to analyse and predict possible solutions to practical problems in the rearing of marine larvae, and likely also later developing stages. Aquaculture needs to become an even more science-based industry. Today a considerable fraction of our knowledge is empirical and system dependent. We can increase the output of knowledge and wisdom from research if we base more of it on theory and fundamental knowledge.

We believe that a paradigm shift in microbial management in aquaculture is needed. Microbes should not be judged collectively as enemies that should be eradicated. Most microbes are beneficial or neutral for hosts, and microbial management should selectively aim at favouring these microbes, instead of a non-selective extinction of microbes. Our hope is that we can go from looking at microbes as enemies to considering them as friends – at least most of them. Thus, we should leave the beat-them strategy and go for the join-them strategy instead. Moreover, microbial management is not about eradication of microbial problems, but about changing probabilities. Consequently, microbial management should involve multiple strategies to improve the probability of mutualistic microbe-larvae interactions (Vadstein et al., 1993b). The implementation of such strategies will result in improved sustainability of the aquaculture industry, including economic sustainability, viability and welfare of fish, and ecological sustainability through reduced use of pharmaceuticals like antibiotics. We conclude that the knowledge on microbial community management by K-selection should be an element in the development of a sustainable microbial management practise in aquaculture.

## Author Contributions

The conceptualization of the study was established and developed by OV and YO, but all the authors were involved in the development of the ideas presented in the manuscript. OV, KA, and IB contributed with the illustrations. OV wrote the first version of the manuscript. The final version of the manuscript was a result of the active participation by all the authors.

## Conflict of Interest Statement

The authors declare that the research was conducted in the absence of any commercial or financial relationships that could be construed as a potential conflict of interest.
